# Adrenal cavernous hemangioma misdiagnosed as pheochromocytoma: a case report

**DOI:** 10.1186/s12893-021-01195-2

**Published:** 2021-04-26

**Authors:** Ting Huang, Qing Yang, Yang Hu, Hai-Xiao Wu

**Affiliations:** Department of Urology, Affiliated Jinhua Hospital, Zhejiang University School of Medicine, Jinhua, 321000 China

**Keywords:** Cavernous, Adrenal hemangioma, Surgical resection, Case report

## Abstract

**Background:**

Adrenal hemangioma is a rare benign adrenal tumor that is usually misdiagnosed preoperatively. We here present a case of adrenal cavernous hemangioma that was successfully treated with retroperitoneal laparoscopic adrenalectomy.

**Case presentation:**

A 67-year-old man with dull right back pain attended our clinic for examination of a mass on the right adrenal gland for 1 week. Pheochromocytoma was considered according to the preoperative computed tomography angiography + computed tomography urography findings and was subsequently corrected to adrenal gland hemangioma according to postoperative pathological findings. The patient showed no recurrence of adrenal hemangioma during the 1-year follow-up period after surgery.

**Conclusion:**

Adrenal gland hemangioma is rare with a high rate of misdiagnosis, and it should be considered in imaging findings of adrenal tumors with typical hemangioma. Surgery is an effective treatment method.

**Supplementary Information:**

The online version contains supplementary material available at 10.1186/s12893-021-01195-2.

## Background

Since the first case report of an adrenal hemangioma published by Johnson [1] in 1955, there have only been 66 cases reported in the literature to date [2]. Adrenal hemangiomas can vary in size, but are usually greater than 5 cm in diameter. These benign masses are hardly ever symptomatic when small, and as the masses increase in size, abdominal or flank pain appear due to the mechanical mass effects on neighboring structures. Most of the reported cases were detected by accident during imaging examinations. They are mostly nonfunctioning tumors, and only 6 clinically functional adrenal hemangiomas have been identified; 3 cases with hyperaldosteronism and the remaining three cases with subclinical Cushing’s syndrome [3–5]. However, some patients with adrenal hemangiomas present with spontaneous life-threatening retroperitoneal hemorrhage [6, 7].

We present the case of an adrenal cavernous hemangioma (63 mm × 95 mm) preoperatively misdiagnosed as pheochromocytoma that was successfully treated by laparoscopic adrenalectomy.

We present the following case in accordance with the CARE reporting checklist (Additional file 1).

## Case presentation

### Chief complaints

On July 12, 2019, a 67-year-old male patient presented to the Affiliated Jinhua Hospital, Zhejiang University School of Medicine (Jinhua, China) because of occasional dull flank pain for 4 years and investigation of a mass on the right adrenal gland for 1 week.

### History of past illness

The patient had suffered from pulmonary nodules for more than 10 years and regular reexamination of these nodules was performed every year.

### Physical examination upon admission

The patient’s height and weight were 168 cm and 52.5 kg, respectively. There were no positive signs in the abdomen or a positive Murphy sign. He had no history of hypertension.

### Laboratory examinations

No abnormal hormone levels were detected during endocrinological examinations, and routine blood tests were within normal limits (plasma renin 7.70 pg/ml, plasma aldosterone 16.4 ng/dl, plasma adrenaline 32 pg/ml, plasma noradrenaline 730 pg/ml).

### Imaging examinations

**Fig. 1:**
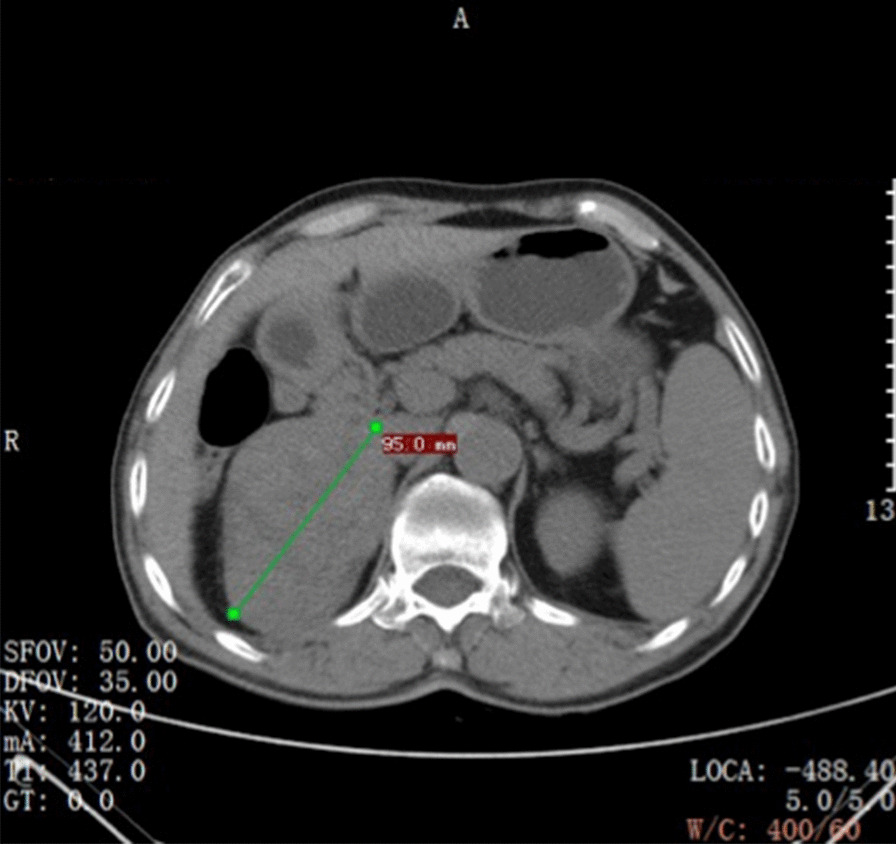
Non-contrast-enhanced CT scan showing an encapsulated large right adrenal lesion with regular margins

**Fig. 2:**
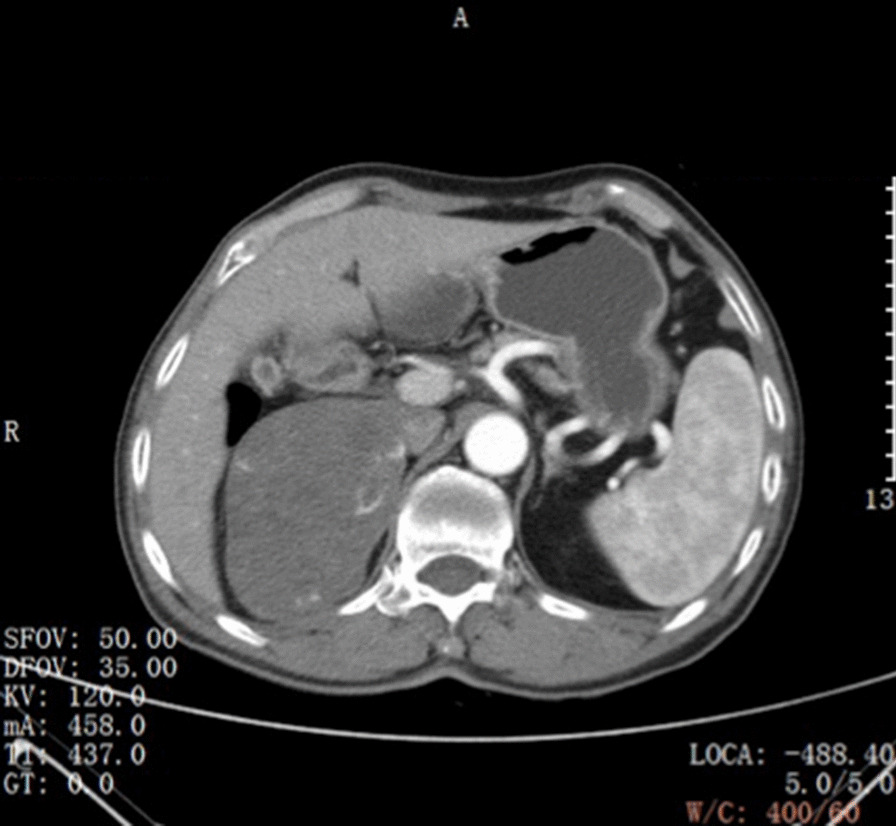
Contrast-enhanced CT images obtained in the arterial phase, 3 min after iodinated contrast administration

**Fig. 3:**
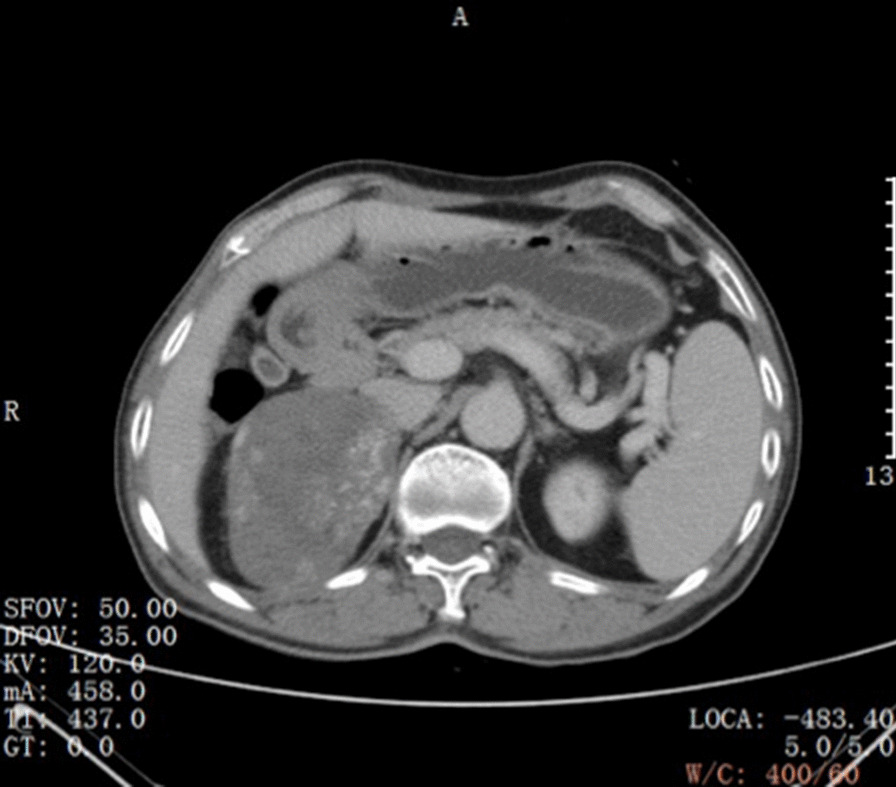
Images obtained 3 min after iodinated contrast administration

**Fig. 4:**
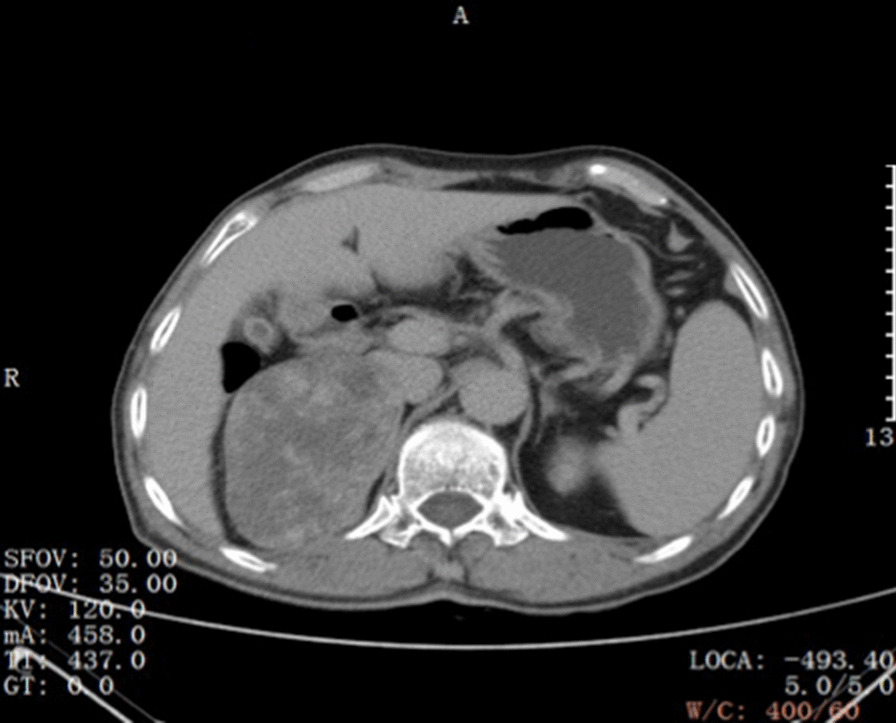
Images obtained 5 min after iodinated contrast administration

Preoperative computed tomography angiography + computed tomography urography (CTA + CTU) showed a mass with a heterogeneous shadow (9.5 × 6.3 cm) in the right adrenal gland (Fig. 1). The mass was well-circumscribed, early peripheral enhancement was obvious in the arterial phase (Fig. 2), with central progressive partial filling in the venous phase and in delayed imaging (Figs. 3, 4).

### Final diagnosis

The patient was diagnosed with an adrenal tumor preoperatively and pheochromocytoma was considered. However, he was subsequently diagnosed with an adrenal cavernous hemangioma.

### Treatment

Retroperitoneal laparoscopic adrenalectomy was performed following the oral administration of Phenoxybenzanine Hydrochloride 10 mg twice a day for one week. Blood volume was fully expanded by fluid infusion two days before surgery to reduce intraoperative blood pressure fluctuations. It took 145 min to complete the operation. Fluctuating or sometimes substantially elevated catecholamine levels in the blood of pheochromocytoma patients can trigger severe cardiovascular complications such as TTS, heart failure, cerebral haemorrhage and sudden cardiac arrest [8] and secondary shock [9]; thus, perioperative monitoring is critical. In this case, the surgeon strictly followed the principle of “minimal touch” during the operation, and intraoperative anaesthetic monitoring showed that the tumour remained stable during intraoperative contact and tumour haemodynamics. Therefore, the patient was not transferred to the ICU for advanced monitoring, but to a general ward with continuous post-operative monitoring of vital signs for 24 h. After determining that their vital signs were stable, the patient was monitored regularly and discharged 6 days after surgery. The patient was discharged 6 d after surgery.

### Pathological findings

**Fig. 5:**
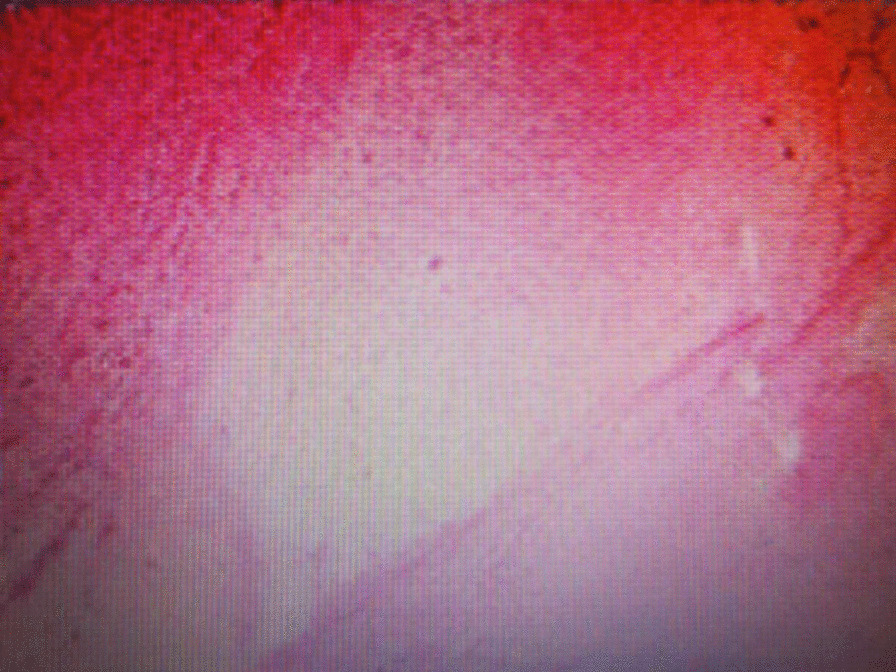
Areas of hemorrhage and necrosis (H&E, ×40)

Histopathologic analysis of the surgical specimen showed an adrenal hemangioma with extensive internal hemorrhage and necrosis (Fig. 5).

### Outcome and follow-up

CT was repeated regularly after surgery. No recurrence, metastasis or other complications were observed after 1 year of follow-up.

## Discussion

Adrenal hemangioma is a rare disease and is difficult to diagnose as no characteristic findings are detected in blood tests or physical examination in most cases. Imaging examinations are important in the diagnosis of adrenal hemangiomas. Ultrasound examinations can be used as a screening tool, but these masses are easily misdiagnosed and the ability of ultrasound to identify adrenal hemangioma and adrenal injury is limited [10]. The hypointense signal can be seen as a streak or patch inside the lesions. The tumour’s solid component of adrenal hemangioma is composed of blood-filled sinusoidsm therefore, imaging manifestations of adrenal hemangioma are similar to that of hemangioma of the liver and spleen in either enhanced MRI or enhanced CT, MRI usually shows slightly hypointense signals on T1-weighted images and hypointensity on T2-weighted images,and there are two characteristic imaging features which may be useful in diagnosing this disease: heterogeneous density on plain scanning and early peripheral enhancement and central progressive partial filling. This Post-enhancement presentation is observed in most adrenal hemangiomas [11] and it is key to the diagnosis. However, Yamada et al. also reported a case that showed only thin marginal enhancement without centripetal enhancement [12]. The imaging features in our case were typical as described above; however, the diagnosis of adrenal hemangioma was not considered before surgery. The imaging features of adrenal hemangiomas should be distinguished from other adrenal lesions [13, 14]. Adrenal cortical adenomas are usually small with clear boundaries, and CT scanning shows hypointense signals and marked enhancement in the arterial phase after contrast medium administration, but low signals in the venous phase. Pheochromocytomas are large lesions with an irregular shape and density, and on contrast-enhanced images sometimes show necrotic patches. Adrenal metastasis can be unilateral or bilateral, with hyperintense signals and enhancement can be seen on contrast-enhanced CT images. The primary malignancies in the cases where the adrenal metastasis were frequently originated from lung cancer, breast cancer and renal cancer [15]. Adrenal cortical carcinomas have boundaries and are irregular in shape, peripheral enhancement can be observed but no enhancement is seen in the center due to hemorrhage and necrosis, and hemorrhage, necrosis and calcification are common. Compared to CT, MRI has the advantage of multi-directional imaging and better resolution of soft tissues, so it has an advantage over CT in determining the origin of tumours and is more valuable in the qualitative diagnosis of tumours. The typical MRI signal features of adenomas resemble the signal intensity of the liver on T1WI and T2WI, with uniform signal intensity. The signal intensity of some tumors are slightly higher than that of the liver on T2WI, and a few tumors have signal intensity similar to that of fat. Adenomas and non-adenomas take on differed enhancement and clearance trends at the enhanced MRI examination: adenomas mostly present early, mild to moderate enhancement with a rapid clearance rate, while non-adenomas chiefly have early to middle, moderate to severe enhancement with a slow clearance rate. Metastatic tumors show a low signal intensity on T1WI and a medium one on T2WI, which is similar to the signal intensity of fat, with uneven signal intensity in most cases, and usually hemorrhage and necrosis areas inside. It has been revealed in studies that adrenal metastases have moderate or significant enhancement on enhanced scanning, some of which develop irregular thick ring enhancement [16].Furthermore, Faria et al. [17] concluded that magnetic resonance spectroscopy (MRS) is a valuable method for identifying adrenal adenomas, pheochromocytomas, adrenal adenocarcinomas and metastases in 60 adrenal tumour cases. It is a pity that MRI was not performed in this case which may provide crucial clues for diagnosis. Along with its notable imaging differences, the adrenal gland is an endocrine organ, and the results of body fluid tests and symptoms associated with hormone secretion can aid the identification of adrenal tumour types. Adrenal adenomas may produce cortisol or aldosterone and may be associated with clinical symptoms such as centripetal obesity, moon-face, hirsutism and hypertension with low blood potassium. Pheochromocytomas are often characterised by persistent or paroxysmal hypertension, palpitations and chest pain. Typically, these symptoms are not treated with standard clinical care due to excessive catecholamine production associated with pheochromocytomas. Measurement of plasma-free norepinephrine and m-adrenaline is the gold standard for the diagnosis of catecholamine hypersecretion [18, 19]. Furthermore, adrenocortical carcinomas may produce several different steroid hormones [20]. In retrospect, there were no significant abnormalities found in the examined body fluid or symptoms associated with abnormal hormone secretion in this case, the primarily imaging-based diagnosis of pheochromocytomas requires further investigation. When we consider the qualitative diagnosis of adrenal masses, we should make good use of available examinations and make a comprehensive consideration as far as possible, especially when no characteristic findings are detected in blood tests or imaging examinations. We reviewed the characteristic of adrenal hemangioma and hope to help clinicians avoid misdiagnosis.

There is no consensus on the management of adrenal hemangiomas. Surgical resection is required if the mass has endocrinological function or pressure-related symptoms appear in order to exclude malignant disease, or is complicated by spontaneous life-threatening hemorrhage. Khalid et al. [21] considered that adrenal incidentalomas larger than 6 cm in diameter should be removed due to the risk of adrenal cancer which is 35% to 98%. Agrusa et al. [22] suggested that adrenal hemangiomas can be treated with conservative measures and periodic follow-up if the tumor is smaller than 3.5 cm and is asymptomatic. In contrast, Wang et al. [23] indicated that adrenal hemangiomas should be resected when detected as the operation is safe and feasible and the capsule can be separated easily. Most adrenal hemangiomas can be managed with laparoscopic surgery; however, open surgery may be considered if the tumor is too large or adrenocortical carcinoma is suspected. Laparoscopic adrenal tumour resection can be performed by either the trans-peritoneal or retroperitoneal route. Both routes have their advantages and disadvantages. The choice of procedure should be based on the patient’s condition, the size and location of the tumour and the surgeon’s practice. The advantages of the transperitoneal approach are the larger operating space and clear anatomical landmarks. At the same time, the retroperitoneal route, although narrower, allows direct access to the surgical target area. It also avoids interference from intraperitoneal organs like the gastrointestinal tract and is more familiar to urologists [24]. In a review of adrenal cavernous hemangioma, AGRUSA [25] found that only a low percentage of cases of adrenal hemangiomas receives a laparoscopic treatment, most cases receives a retroperitoneal treatment. While it has become the mainstream procedure for adrenal tumours, tumour separation during lumpectomies for large adrenal tumours have more significant space requirements. The transabdominal approach was chosen as it reduces the obstruction of the surgical field, lumpectomy interference, and speeds up the surgical process, thus facilitating a smooth operation.

## Conclusion

Adrenal hemangiomas are benign nonendocrine lesions with complete capsules and are rarely recurrent or malignant; thus, the prognosis of patients with these lesions is satisfactory. Imaging examinations are valuable in preoperative diagnosis. Knowledge of these lesions can avoid misdiagnosis. Surgical resection is an effective treatment strategy.

## Supplementary Information

**Additional file 1:** Imaging examinations information and pathological findings.

## Data Availability

The datasets used during this study available from the corresponding author on reasonable request.

## Abbreviations

CT: Computed tomography
CTA: Computed tomography angiography
CTU: Computed tomography urography
MRI: Magnetic resonance imaging
MRS: Magnetic resonance spectroscopy

## References

[1] Johnson CC, Jeppesen FB (1955). Hemangioma of the adrenal. J Urol..

[2] Degheili JA, Abou HNF, El-Moussawi M (2019). Adrenal cavernous hemangioma: a rarely perceived pathology-case illustration and review of literature. Case Rep Pathol.

[3] Oishi M, Ueda S, Honjo S (2012). Adrenal cavernous hemangioma with subclinical Cushing’s syndrome: report of a case [J]. Surg Today.

[4] Lorenzon L, Ziparo V, Caterino S (2013). Bilateral cavernous hemangiomas of the adrenal glands presentation and management of an unusual incidental finding. Ann Ital Chir.

[5] Stumvoll M, Fritsche A, Wehrmann M, Dammann F, Becker HD, Eggstein M (1996). A functioning adrenocortical hemangioma. J Urol.

[6] Thomas L, Forbes MD (2005). Retroperitoneal hemorrhage secondary to a ruptured cavernous hemangioma. Can J Surg.

[7] Piotr P, Iwona A, Katarzyna HD (2010). Spontaneous rupture of adrenal haemangioma mimicking abdominal aortic aneurysm rupture. Arch Med Sci.

[8] Yuan S, He T, Yang L (2020). Basal Takotsubo syndrome induced by pheochromocytoma rupture. Cardiovasc J Afr.

[9] Maestroni U, Ziglioli F, Baciarello M (2019). Multidisciplinary management of a large pheochromocytoma presenting with cardiogenic shock: a case report. BMC Urol.

[10] Pang C, Wu P, Zhu G (2015). A rare cavernous hemangioma of the adrenal gland. Urol Case Rep.

[11] Xu HX, Liu GJ (2003). Huge cavernous hemangioma of the adrenal gland: sonographic, computed tomographic, and magnetic resonance imaging findings. J Ultrasound Med.

[12] Yamada T, Ishibashi T, Saito H (2002). Two cases of adrenal hemangioma: CT and MRI findings with pathological correlations. Radiat Med.

[13] Pailin L, Malai M, Pannee V (2004). Imaging features of unusual adrenal masses. Australas Radiol.

[14] Lattin GE, Sturgill ED, Tujo CA (2014). From the radiologic pathology archives: Adrenal tumors and tumor-like conditions in the adult: radiologic-pathologic correlation. Radiographics..

[15] Tsujimoto A, Ueda T, Kuge H (2019). Long-term survival after ad- renal metastasectomy from colorectal cancer: a report of two cases. Surg Case Rep.

[16] Hönigschnabl S, Gallo S, Niederle B (2002). How accurate is MR imaging in characterisation of adrenal masses: update of a long-term study. Eur J Radiol.

[17] Faria JF, Goldman SM, Szejnfeld J (2007). Adrenal masses: characterization with in vivo proton MR spectroscopy–initial experience. Radiology.

[18] Pacak K, Linehan WM, Eisenhofer G (2001). Recent advances in genetics, diagnosis, localization, and treatment of pheochromocytoma. Ann Intern Med.

[19] Chen H, Sippel RS, ODorisio MS, et al; North American Neuroendocrine Tumor Society (NANETS). The North American Neuroendocrine Tumor Society consensus guideline for the diagnosis and management of neuroendocrine tumors: pheochromocytoma, paraganglioma, and medullary thyroid cancer. Pancreas. 2010;39(6):775–83. 10.1097/MPA.0b013e3181ebb4f0 PMC3419007 20664475

[20] Libè R, Fratticci A, Bertherat J (2007). Adrenocortical cancer: pathophysiology and clinical management. Endocr Relat Cancer.

[21] Khalid SA, Bokhari SA, Alkeraithi M (2011). Adrenal hemangioma in a 19-year-old female. Ann Saudi Med..

[22] Agrusa A, Romano G, Salamone G (2015). Large cavernous hemangioma of the adrenal gland: laparoscopic treatment. Report of a case. Int J Surg Case Rep..

[23] Wang C, Feng L, Wang L (2014). The left giant adrenal hemangioma(a report of one case and literature review). J Mod Urol..

[24] Vrielink OM, Wevers KP, Kist JW (2017). Laparoscopic anterior versus endoscopic posterior approach for adrenalectomy: a shift to a new golden standard?. Langenbecks Arch Surg.

[25] Agrusa A, Romano G, Dominguez LJ (2016). Adrenal cavernous hemangioma: which correct decision making process?. Acta Medica Mediterranea.

